# By the Way

**Published:** 1910-08-06

**Authors:** 


### By the Way.

### THE REPRESSION OF QUACKS.

We are indebted to *La Belgique Médicale* for the following comments on a report of a recent meeting of the Municipal Council taken from a Parisian daily: "M. Salmon declared that not only did certain drug-shops, pompously styled 'Universities,' 'Institutes,' etc., defraud the Treasury by displaying advertisements upon which no tax had been paid, but that in addition they compromised the health, such as it was, of their artless clients. He therefore demanded that the Treasury should prosecute individuals connected with these places, if solvent, and, if not, that the establishments should be forcibly closed. In the second place, he asked that such doings of charlatans as were dangerous to public health should be vigorously repressed. M. Laurent, General Secretary to the Prefecture of Police, replied that prosecutions were already taking place, and that in future greater severity would be shown. The Council, while accepting these assurances, demanded that similar repressive measures should be directed against certain midwives, who were in reality nothing more nor less than 'faiseuses d'anges' (angel-makers)." The example given by the Paris ædiles is one to be called attention to and one to be imitated. Every year a greater invasion of the large towns occurs owing to the redoubtable enterprise of quack doctors and barefaced abortionists. When, exclaims our contemporary, will we make a clean sweep of this sinister bear-garden?

### BATH WATERS.

Last week's visit of a party of medical men to Bath draws attention to the fact that the waters there are often referred to by old English writers on medical topics. An extract from Dr. Arthur E. W. Fox of Bath's "Manual of the Bath Mineral Waters," published in 1888, may be of interest. After reference to the beneficial use of the waters after attacks of rheumatic fever, he adds: "It is, however, in chronic rheumatism that the Bath waters are especially to be considered worthy of trial. In cases where the joints are swollen, painful, and stiff, also in lumbago, sciatica, and neuralgic rheumatism, the water administered internally and the baths at a carefully regulated temperature, either Aix douche or general immersion, combined with a gentle undercurrent douche, prove most beneficial. During the past thirteen years we have had under our care at Bellot's Hospital a large number of cases of chronic rheumatism, and can therefore conscientiously state that in by far the majority of these cases a marked and permanent relief has ensued from purely thermal treatment in the shape of immersion baths, combined with douching and the internal use of the waters. In the treatment of chronic synovitis, whether of rheumatic, gouty, or strumous origin, the Bath waters have long been regarded as of great value both in the early and late stages.

